# Radioprotective and Antioxidant Effect of Resveratrol in Hippocampus by Activating Sirt1

**DOI:** 10.3390/ijms15045928

**Published:** 2014-04-09

**Authors:** Jianguo Li, Li Feng, Yonghua Xing, Yan Wang, Liqing Du, Chang Xu, Jia Cao, Qin Wang, Saijun Fan, Qiang Liu, Feiyue Fan

**Affiliations:** 1 Tianjin Key Lab of Molecular Nuclear Medicine, Institute of Radiation Medicine of Chinese Academy of Medical Science and Peking Union Medical College, Tianjin 300192, China; E-Mails: ljg7111@126.com (J.L.); 1318007143@126.com (L.F.); foreverjian71@163.com (Y.X.); xueqiongtongliao@126.com (Y.W.); jianguotongliao@126.com (L.D.); xuchangtj@126.com (C.X.); caojiatj@126.com (J.C.); jian711123@126.com (Q.W.); yangjiejiehouqi@126.com (S.F.); 2 Department of Human Anatomy, The Medical School of Inner Mongolia University for the Nationalities, Tongliao 028041, Neimenggu, China

**Keywords:** resveratrol, Sirtuin 1, radiation, reactive oxygen species, hippocampus

## Abstract

Reactive oxygen species can lead to functional alterations in lipids, proteins, and nucleic acids, and an accumulation of ROS (Reactive oxygen species) is considered to be one factor that contributes to neurodegenerative changes. An increase in ROS production occurs following irradiation. Neuronal tissue is susceptible to oxidative stress because of its high oxygen consumption and modest antioxidant defenses. As a polyphenolic compound, resveratrol is frequently used as an activator of Sirt1 (Sirtuin 1). The present study was designed to explore the radioprotective and antioxidant effect of resveratrol on Sirt1 expression and activity induced by radiation and to provide a new target for the development of radiation protection drugs. Our results demonstrate that resveratrol inhibits apoptosis induced by radiation via the activation of Sirt1. We demonstrated an increase in Sirt1 mRNA that was present on 21 days of resveratrol treatment following irradiation in a concentration-dependent manner. Such mRNA increase was accompanied by an increase of Sirt1 protein and activity. Resveratrol effectively antagonized oxidation induced by irradiation, supporting its cellular ROS-scavenging effect. These results provide evidence that the mitochondrial protection and the antioxidant effect of resveratrol contribute to metabolic activity. These data suggest that Sirt1 may play an important role to protect neurons from oxidative stress.

## 1. Introduction

Radiation therapy represents a targeted, non-invasive and potentially organ-preserving therapy. It has been recognized that radiation-induced effects remain a significant risk. Protection of biological systems from ionizing radiation is of paramount importance in planned as well as unplanned accidental exposures to radiation [1,2], and the development of novel and effective agents to combat radiation damages using nontoxic radioprotectors is of considerable interest in defense, nuclear industry, space travels, and health care, particularly in radiodiagnostics and therapy. Many synthetic as well as natural compounds have been investigated the recent past for their efficacy to protect the biological systems against the deleterious effects of radiations. They include sulfhydryl compounds, antioxidants, plant extracts, immunomodulators, and other agents [3,4]. Reactive oxygen species (ROS), in particular the hydroxyl radical, can lead to functional alterations in lipids, proteins, and nucleic acids, and an accumulation of ROS is considered to be one factor that contributes to neurodegenerative changes, for example in Parkinson’s disease [5] and Alzheimer’s disease [6]. An increase in ROS production occurs following irradiation [7], and therefore, it is not surprising to find that at least some effects of exposure of cells to ROS are mimicked by exposure of cells to ionizing radiation. Neuronal tissue is susceptible to oxidative stress because of its high oxygen consumption and modest antioxidant defenses [8].

The underlying mechanisms of this injury remain unclear, however there is an increasing amount of data indicating that the response of the central nervous system (CNS) after radiotherapy is a continuous, dynamic, and interactive process [9,10]. At the cellular level, Sirt1 is present in both the nucleus and cytoplasm with dominant expression in the nucleus. Sirt1 has a significant role in multiple biological processes that include oxidativestress, metabolism, cellular proliferation and genomic stability. Interestingly, Sirt1 has been demonstrated to regulate cellular protection against oxidative stress in many disease states that involve neurodegeneration, metabolic disorders and cardiovascular disease [11].

Resveratrol (RSV; 3,4,5-trihydroxy-trans-stilbene) is a natural non-flavonoid polyphenolic found in the skin of red grapes [12]. Many studies have shown that resveratrol can prevent or slow the progression of a variety of conditions, including cancers, cardiovascular diseases, or ischemic injuries, and can enhance stress resistance and extend lifespan [13,14]. As a polyphenolic compound, resveratrol is frequently used as an activator of Sirt1; it has also been shown to be a scavenger of hydroxyl, superoxide, and metal-induced radicals [15,16]. Recently, mice given resveratrol before radiation were shown to have increased survival rates [17]. It is unknown whether resveratrol activates Sirt1 to anti-apoptosis induced by radiation in hippocampus, and it is also unknown which intracellular signaling pathways contribute to this phenomenon. In our study, we propose that resveratrol exerts radioprotective and antioxidant effects by activating Sirt1.

## 2. Results and Discussion

### 2.1. Immunohistology and TUNEL-Positive Cells within the Hippocampi

**Figure 1.**
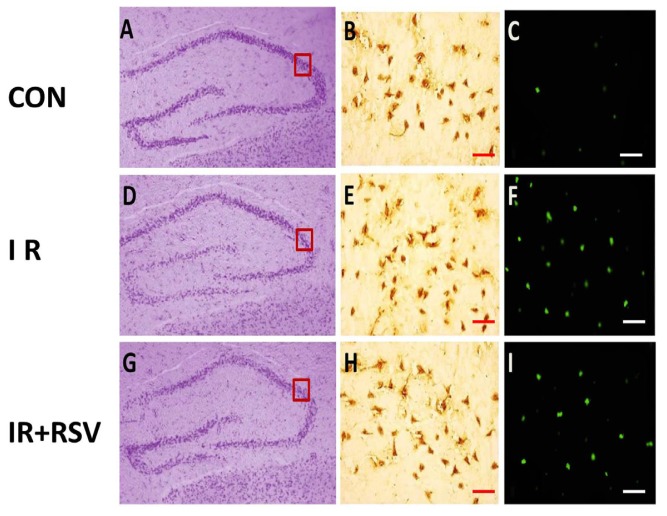
Representative micrographs of the hippocampal formation from coronal brain sections taken from rats of con group, IR group and IR + RSV group 24 h after irradiation. Sections in CA3 subfield of hippocampus were stained either with CV (**A**,**D**,**G**), Immunohistology of Sirt1 (**B**,**E**,**H**) or stained with TUNEL (**C**,**F**,**I**). CV staining showed widespread damage of pyramidal neurons apparent with disrupted Nissl staining pattern in CA3 of the IR group (**D**) and IR + RSV group (**G**) compared with con group (**A**). Quantification of Sirt1 protein expression in CA3 subfield of hippocampus immunohistochemically stained with anti-Sirt1 antibody according to the ABC method. The levels of Sirt1 present in the hippocampi following irradiation (IR group and IR + RSV group; (**E**,**H**) were significantly increased compared with the normal control (**B**). TUNEL-positive cells were visible mainly in the hippocampi of the IR group (**E**), next visible in the IR + RSV group (**H**). Compared with the other groups, the number of TUNEL-positive cells detected in the control rats was low (**C**). Scale bars: (**B**,**C**,**E**,**F**,**H**,**I**) 50 μm. The box indicates the image positioning of immunohistology and TUNEL. CV, Cresyl Violet; TUNEL, terminal deoxynucleotidyl transferase dUTP nick end-labeling; Sirt1, Sirtuin 1; IR, irradiation; RSV, Resveratrol.

Sirt1 was mainly expressed with positive yellow-brown staining and a high concentration of brown granules (Figure 1B,E,H). In the hippocampi of the normal control group, Sirt1 was predominantly expressed in the present in both the nucleus and cytoplasm of neurons (Figure 1B). The levels of Sirt1 present in the hippocampi following irradiation (IR group and IR + RSV group; Figure 1E,H) were significantly increased compared with the normal control. TUNEL-positive cells were visible mainly in the hippocampi of the IR group (Figure 1F), next visible in the IR + RSV group (Figure 1I). Compared with the other two groups, the number of TUNEL-positive cells detected in the control rats was low (Figure 1C).

### 2.2. Neuroprotective Effects of Resveratrol

**Figure 2.**
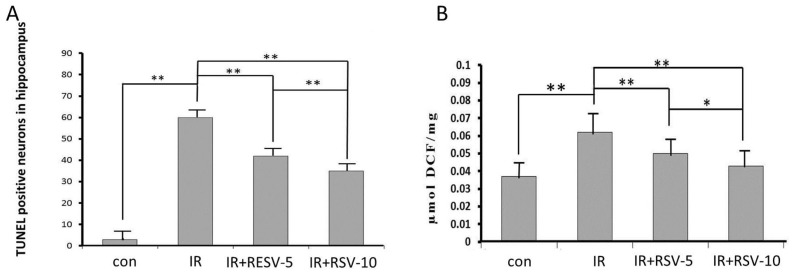
TUNEL staining (**A**) and mean values for ROS production (**B**). TUNEL staining shows positive neurons in the hippocampi. The neurons in hippocampi from control rats display few positive neurons. Radiated rats remarkably become evident by a prominent growth in the number of TUNEL positive cells. RSV administration on TUNEL positive neurons following radiation remarkably decreased compared with only radiated. Irradiation significantly increased ROS production in IR group compared with rats of control group. Treatment with RSV reversed the irradiation-induced change in IR + RSV group compared with IR group in a concentration-dependent manner. * *p* < 0.05, ** *p* < 0.01. DCF, dichlorofluorescein.

Widespread damage of pyramidal neurons apparent with disrupted Nissl staining pattern in CA3 of the IR and IR + RSV groups 24 h following irradiation. The quantification of TUNEL-positive neurons 24 h after exposure to radiation in the hippocampi indicated the neuroprotective effects of Sirt1. The number of TUNEL-positive neurons observed in the hippocampi of the IR + RSV group following irradiation was reduced compared with that of the IR group (Figure 2A).

### 2.3. Sirt1 Expression by Western Blot and qRTPCR

**Figure 3.**
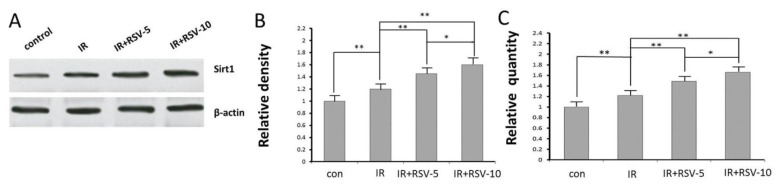
Effects of RSV on the expression levels of Sirt1. (**A**) Western blot analysis representative pattern from six rats, (**B**) protein expression levels and (**C**) mRNA expression levels of Sirt1 are expressed as relative density data which are the mean ± standard deviation from six rats in two independent experiments. The expression of Sirt1 protein was significantly enhanced in hippocampal tissue prepared from rats of IR group compared with rats of control group (**A** and **B**). Treatment with RSV reversed the irradiation-induced change in IR + RSV group compared with IR group (**A** and **B**). Sirt1 mRNA was significantly upregulated in IR group, compared with the control group. Consistently, Sirt1 mRNA was noticeably lower in IR group than that in IR + RSV group in a concentration-dependent manner (**C**).* *p* < 0.05, ** *p* < 0.01. IR, irradiation.

The expression of Sirt1 protein was significantly enhanced in hippocampal tissue prepared from rats of IR group compared with rats of control group (Figure 3A,B). Treatment with RSV reversed the irradiation-induced change in IR + RSV group compared with IR group (Figure 3A,B). Sirt1 mRNA was significantly upregulated in IR group compared with the control group. Consistently, Sirt1 mRNA was noticeably lower in IR group than that in IR + RSV group (Figure 3C).

### 2.4. Mean ROS Accumulation

It was significantly enhanced in hippocampal tissue prepared from rats of IR group compared with rats of control group (Figure 2B). Treatment with RSV reversed the irradiation-induced change in IR + RSV group compared with IR group (Figure 2B).

### 2.5. Sirt1 Activity after IR and Resveratrol Treatment

**Figure 4.**
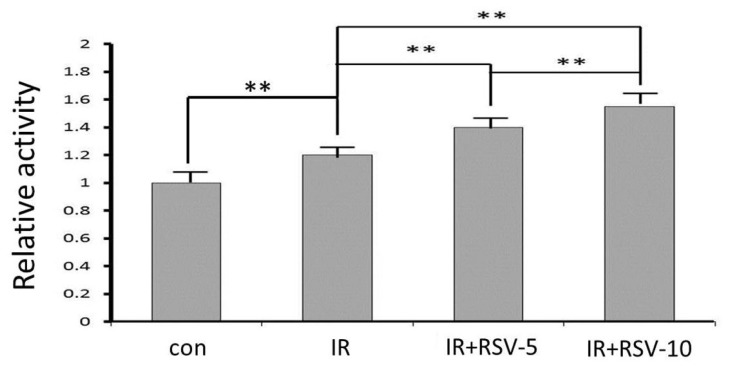
Sirt1 enzyme activity after IR and resveratrol treatment. Activity of Sirt1 enzyme was measured in total protein extracts from hippocampal tissues of rats subjected to RSV or IR procedure. Results were normalized to control levels, and the significance was calculated by the unpaired *t* test with the Welch correction. ** *p* < 0.01.

To determine the mediators of mitochondrial function after IR, Sirt1 activity was measured, and the results demonstrated a significant increase after IR (Figure 4). Resveratrol treatment along with IR increased Sirt1 activity. Resveratrol enhanced the Sirt1 activity in a concentration-dependent manner (Figure 4).

Sirt1 provides cells with tolerance against oxidative stress. In some cells, Sirt1 may offer protection against oxidative stress through the modulation of fork head transcription factor. Sirt1 also protects cells against oxidative stress by increasing the activity of catalase [18]. Sirt1 overexpression enhances the tolerance against free radical toxicity in neuronal cells [19,20]. Sirt1 can block p53-induced apoptosis through p53 deacetylation and induction of manganese SOD (MnSOD) [21,22]. In many experimental paradigms, resveratrol, a naturally occurring phytoalexin polyphenol in grapes and red wine, is used to increase Sirt1 activity. Resveratrol treatment prevents apoptotic injury in vascular endothelial cells during models of experimental diabetes with elevated glucose [23]. In contrast, inhibition of Sirt1, such as with nicotinamide, can block proliferation and lead to apoptosis in leukemic cells, possibly through p53-dependent and independent mechanisms [24]. Furthermore, agents such as Sirtinol that inhibit Sirt1 activity can be detrimental to neurons during oxidative stress [25] while the use of the specific small-molecule inhibitor of Sirt1 EX527 [26] can block HDAC activity and increase vascular injury during oxidative stress, suggesting that an endogenous level of Sirt1 is required for vascular protection [23].

Resveratrol is a type of polyphenol and an antimicrobial substance synthesized de novo by plants (a phytoalexin). Resveratrol is found in the skin of red grapes and is a component of red wine [24]. The other sources of resveratrol include raspberries, mulberries, plums, peanuts, bilberries, blueberries, cranberries, Scots pine and Japanese knotweed. Resveratrol is synthesized instinctively by the above plants as a protection to counter the bacterial and fungal infections, stress and injury [25]. Resveratrol received substantial notice with the emergence of the “French paradox”, which is portrayed by the reduced prevalence of cardiovascular diseases in the red wine-drinking southern French population notwithstanding eating foods that are rich in saturated fats [26]. Although resveratrol subsists as both *cis*- and transisomeric-forms, the transisomer is the steady form of resveratrol, which is also the isomer that plays a role in nearly all biological actions of resveratrol [27]. Resveratrol mediates a variety of biological activities which comprise extension of the life span even when fed a high caloric diet and cancer prevention [28]. Studies in animal models also imply a number of other beneficial health effects of resveratrol, which comprise anti-ischemic, antiviral, antioxidant and anti-inflammatory properties [29,30].

Resveratrol modulates the synthesis of lipids, lipid catabolism, and apoptosis, and it possesses anti-cancer and anti-inflammatory properties [31]. Ogawa *et al.* reported that mice given resveratrol before radiation had significantly higher survival rates, which is due, at least in part, to resveratrol’s regulation of superoxide dismutase and glutathione peroxidase [32]. Moreover, Şimşek *et al.* reported that resveratrol could ameliorate salivary gland and ovarian damage induced by radiation [33]. Mudò *et al.* reported resveratrol acting via Sirt1/PGC-1α may prove useful as neuroprotective agents in PD and possibly in other neurological disorders [34]. Our experiments now demonstrate that Sirt1 activity is significantly increased after IR. This observation is consistent with our studies that have shown a significant increase in the expression of total cellular Sirt1 protein after IR. Resveratrol treatment augmented the expression and activity of Sirt1. Resveratrol is a known allosteric activator of Sirt1, therefore, the mechanism of its action likely involves activation of Sirt1 enzyme activity [35,36]. It is now clear from our experimental results that there is a functional boost of Sirt1 after IR, and resveratrol treatment was able to increase activity and expression of Sirt1 in a concentration-dependent manner. However, it is still unresolved whether resveratrol is modulating other critical genes. Sirt1 levels can increase but the protective effect of resveratrol may be Sirt1-independent. Future experiments using Sirt1 knockout mice may show the real correlation between Sirt1 and resveratrol.

## 3. Experimental Section

### 3.1. Chemicals and Animals

Resveratrol (3,4,5-trihydroxy-trans-stilbene) was purchased from Sigma Chemicals (St. Louis, MO, USA). All animal procedures were performed in a facility accredited by the Radiation Hazard Evaluation Laboratory of the Institute of Radiation Medicine of Chinese Academy of Medical Science and Peking Union Medical College (Nankai, Tianjing, China). All experimental procedures were performed according to the Guide for the Care and Use of Laboratory Animals published by the US National Institutes of Health (publication no. 85-23, revised 1996). Male Sprague-Dawley rats weighing 200–220 g were randomly divided into four groups (12 rats/group): the irradiation group (IR group), the irradiation with resveratrol 5 mg/kg group (IR + RSV-5 group), the irradiation with resveratrol 10 mg/kg group (IR + RSV-10 group) and control group (con group).

### 3.2. Drug Treatments

Rats in control group and IR group were given distilled water, while in IR + RSV-5 and IR + RSV-10 groups were respectively given drinking water plus 5 and 10 mg/kg resveratrol for 21 days.

### 3.3. Radiation Model

The irradiation of the rats in IR and IR + RSV groups was performed at room temperature using a Cs-137 γ-ray instrument (Atomic Energy of Canadian Inc., Mississauga, ON, Canada) to administer a 4-Gy dose of radiation at a dose rate of 0.71116 Gy/min. The animals in the control group did not receive any radiation. The study was reviewed and approved by the Institutional Animal Care and Use Committee (IACUC) of Institute of Radiation Medicine of Chinese Academy of Medical Science and Peking union Medical College (Tianjin, China). Twenty-four hours subsequent to irradiation, the rats from each group were anesthetized with 10% chloral hydrate (30 mg/kg body weight) by intraperitoneal anesthesia.

### 3.4. Terminal Deoxynucleotidyl Transferase dUTP Nick End-Labeling (TUNEL) Staining, Immunohistology and Cresyl Violet (CV) Staining

Rat brains were harvested and immediately frozen in 2-methylbutane at −30 °C. Coronal sections were cut into 12-μm thick sections with a cryostat (CM 3000; Leica, Manheim, Germany) at the level of the CA3 subfield of hippocampus and then stored at −80 °C until required for further experiments. Coronal sections were air dried for 15 min, post-fixed in 10% formalin for 15 min, washed twice in PBS and then processed for immunohistology with rabbit anti-Sirt1 (1:1000 dilution; Abcam, Cambridge, MA, USA). The avidin-biotin-peroxidase complex method was conducted as previously described [37]. For detection of DNA fragmentation, the fluorescein-based TUNEL assay (Roche Molecular Biochemicals, Indianapolis, IN, USA) was used. TUNEL staining was conducted according to the manufacturer’s instructions. Briefly, sections were incubated for 90 min at 37 °C with TUNEL reaction mixture. Positive control sections were incubated with 200 U/mL DNase I (Gibco-BRL, Carlsbad, CA, USA) for 5 min prior to fixation. Negative control sections underwent the same procedure but terminal deoxynucleotidyl transferase was omitted from the reaction buffer to evaluate nonspecific labeling. TUNEL cell counts were performed on brain sections (*n* = 6) from the hippocampi. TUNEL-positive cells were averaged from counts on three adjacent brain sections of a rat. Images were visualized using a Leica microscope under an excitation/emission wavelength of 500/550 nm (green), captured using an Optronics DEI-750 3-chip camera equipped with a BQ 8000 sVGA frame grabber and analyzed with Bioquant software (Bioquant Image Analysis Corporation, Nashville, TN, USA). The sections were stained with Cresyl Violet (CV) using the conventional method and mounted.

### 3.5. Western Blot Analysis

Animals were euthanized at 24 h following irradiation, and hippocampi (*n* = 6 each group) were obtained. The total protein and nuclear protein were isolated from hippocampi using RIPA buffer (Beyotime, Jiangsu, China) according to the manufacturer’s instructions. The protein concentration from the cytosol (supernatant) was determined spectrophotometrically from the absorbance at 595 nm (A595 nm) using the Hokari method [38]. Samples containing equal amounts of protein were mixed with loading buffer with 5% 2-mercaptoethanol, heated for 5 min at 95 °C, loaded onto a 10% SDS-PAGE gel, and transferred to polyvinylidene difluoride membranes (Millipore, Billerica, MA, USA). After blocking with 5% milk and 0.1% Tween-20 in Tris-buffered saline (TBS), membranes were incubated overnight at 4 °C with the following primary antibodies: rabbit anti-Sirt1 antibody which was obtained from Cell Signaling Technology (1:500 dilution; Beverly, MA, USA). Rabbit anti-β-actin (1:1500 dilution; Sangon Biotech, Shanghai, China) and goat anti-rabbit IgG conjugated to horseradish peroxidase (1:500 dilution; ZSGB-BIO, Beijing, China).

### 3.6. RNA Extraction, cDNA Synthesis, and Quantitative Real-Time PCR (qRT-PCR)

Total RNA was purified and extracted as conducted previously by Chen *et al.* [39]. Equal concentrations of total RNA were reverse-transcribed using Prime Script RT reagent kit (Takara Bio, Inc., Shiga, Japan) according to the manufacturer’s instructions. cDNA samples were blended with primers and SYBR Master Mix (Invitrogen Life Technologies, Carlsbad, CA, USA) in a total volume of 25 μL. All samples were assayed in triplicate using an ABI PRISM 7500 Sequence Detection system (Applied Biosystems^®^-Life Technologies, Foster City, CA, USA). The cycle threshold (CT) values for each reaction were determined and the mean was calculated using TaqMan SDS analysis software (Applied Biosystems^®^-Life Technologies, Foster City, CA, USA). The expression levels of target genes were calculated by the comparative *C*_t_ method (fold changes = 2^(−ΔΔ^*^C^*^t)^). PCR primers for Sirt1 were obtained from Sangon Biotech (Shanghai, China). The primer pairs used were as follows: Sirt1, 5′-CCAGATCCTCAAGCCATGT-3′ (forward) and 5′-TTGGATTCCTGCAACCTG-3′ (reverse) [40].

### 3.7. Analysis of Reactive Oxygen Species

The formation of reactive oxygen species was assessed in the impure synaptosomal preparation, P_2_, as described previously [41]. The method based on oxidation of the non-fluorescent probe, 2 Carlsbad, CA, 7′-dichlorofluorescin diacetate, by reactive oxygen species, to the highly fluorescent 2′,7′-dichlorofluorescein. To assess reactive oxygen species production, synaptosomes were prepared as described previously [42] from hippocampal slices obtained from rats of each group. The resultant pellet was resuspended in 1 mL of ice-cold 40 mM Tris buffer (pH 7.4), and aliquots (1 mL) of homogenate were incubated with 2′,7′-dichlorofluorescin diacetate (10 μL; final concentration 5 μM; from a stock solution of 500 μM Min methanol; Molecular Probes, Eugene, OR, USA) at 37 °C for 15 min. To terminate the reaction, the dye-loaded synaptosomes were centrifuged at 13,000× *g* for 8 min. The pellet was resuspended in 1.5 mL of ice-cold 40 mM Tris buffer (pH 7.4), and fluorescence was monitored at a constant temperature of 37 °C at 488 nm excitation (bandwidth 5 nm) and 525 nm emission (bandwidth 20 nm). Reactive oxygen species formation was quantified from a standard curve of 2′,7′-dichlorofluorescein in methanol (range 0.05 to 1 μM). Results were expressed as nmol/mg tissue corrected for protein.

### 3.8. Sirt1 Activity Assay

The enzymatic activity of Sirt1 in the hippocampal tissue was assayed by a fluorimetric assay by using the SensoLyte Green Sirt1 assay kit (AnaSpec, Fremont, CA, USA). This assay was appropriate for the study, since our objective was to test hippocampal tissue Sirt1 activity, and was performed according to the manufacturer’s directions. The acetylated p53 peptide substrate provided with the kit was incubated with Sirtuin containing tissue protein samples [22]. Deacetylation of substrate sensitizes it to the color developer releasing the green fluorophore. The fluorescence signal generated was in proportion to the amount of deacetylation of the lysine.

### 3.9. Statistical Analysis

Data are presented as the mean ± standard deviation. Data were analyzed using one-way analysis of variance with a *post hoc* test (multiple comparison test), which determined the significant differences among groups. *p* < 0.05 was considered to indicate a statistically significant difference.

## 4. Conclusions

In conclusion, our results demonstrate that resveratrol inhibits apoptosis induced by radiation via the activation of Sirt1. We demonstrated an increase in Sirt1 mRNA that was present on 21 days of resveratrol treatment. Such mRNA increase was accompanied by an increase of Sirt1 protein and activity. In the present study, resveratrol effectively antagonized oxidation induced by irradiation, supporting its cellular ROS-scavenging effect. These results provide evidence that the mitochondrial protection and the antioxidant effect of resveratrol contribute to metabolic activity. These data suggest that Sirt1 may play an important role to protect neurons from oxidative stress.
